# Comparison of gut microbiota immunity and pathology in specific-pathogen-free chickens with glandular and muscular gastritis using different methods

**DOI:** 10.3389/fvets.2024.1343768

**Published:** 2024-06-03

**Authors:** Zunxiang Yan, Shifa Yang, Shuqian Lin, Zengcheng Zhao, Yueyue Liu, Bin Yin, Yunpeng Yi, Shikai Song, Rongling Zhang, Zhongli Huang

**Affiliations:** Shandong Provincial Animal and Poultry Green Health Products Creation Engineering Laboratory, Institute of Poultry Science, Shandong Academy of Agricultural Science, Jinan, China

**Keywords:** glandular and muscular gastritis, SPF chicken, animal model, 16S rDNA, gut microbiome

## Abstract

The objective of this study is to review different methods to screen for the optimal model for preventing and treating chicken glandular and muscular gastritis syndrome. Twenty-four 40-day-old specific pathogen-free (SPF) chickens were randomly allocated into four groups (*N* = 6): polyethylene glycol + ammonium chloride group (M1 group), acetic acid + rhubarb group (M2 group), polyethylene glycol + rhubarb group (M3 group), and control group. The control group had free access to water, while the remaining groups received different doses of molding reagents added to their drinking water. The animal models were assessed based on clinical manifestations, histopathology findings, serological analysis, and composition of intestinal microbiota to establish an optimal approach for constructing an avian model of glandular and muscular gastritis. The SPF chickens in each model group exhibited typical symptoms of glandular and muscular gastritis, poor spirit, yellow loose stools with undigested feed, and enlargement and ulceration of the glandular and muscular stomach. Among these groups, the M3 group had the highest incidence rate of 100%. Compared to the control group, the body weight and body temperature of the chicken in the three model groups were reduced, and the glandular and muscular stomachs and duodenum showed different degrees of bleeding, mucosal abscission, and other pathological injuries. Additionally, the levels of serum IL-2 and α-amylase activity decreased while the content of IL-4 increased. After conducting 16s rDNA sequencing, it was observed that the abundance of *Bacteroides, Faecalibacterium*, and *Ruminococcaceae UCG-014* was significantly increased in the model group compared to the control group. Conversely, there was a notable decrease in the levels of *Megamonas* and *Lactobacillus*, which are speculated to be associated with arachidonic acid metabolism, the NF-κB signaling pathway, and TNF signaling pathways. The combination of polyethylene glycol and rhubarb emerged as the most effective method for establishing the glandular and muscular gastritis model in SPF chickens. This constructed chicken model displayed distinct signs of damage to the glandular and muscular stomach, inflammatory response, and disturbance in the intestinal flora, thereby providing a foundation for future research on the prevention and treatment of this syndrome.

## Introduction

Gastrointestinal health issues in chickens have been a significant impediment to the rapid progress of poultry breeding, with glandular and muscular gastritis increasingly prevalent in poultry farming (1). This disease can lead to enlargement of the glandular and muscular stomachs, as well as other pathological changes associated with gastritis. At autopsy, chickens affected by this condition exhibit notable enlargement of the glandular and muscular stomachs, flattening and disappearance of gastric papillae, and ulceration of the gastric mucosa. In severe cases, it can also present with symptoms such as gastric mucosal bleeding (2). When the muscular and glandular stomachs of chickens suffer from pathological damage, it can lead to disturbances in the digestive system and subsequent dysfunction throughout the body. The main symptoms of sick chickens are sluggish growth, obvious emaciation, decreased feed utilization efficiency, yellow watery feces, and some feces containing undigested feed (3).

There is no obvious seasonality and regionality in the occurrence of glandular and muscular gastritis, and it affects chickens nationwide throughout the year. The etiology of this disease is complex, and there is no accurate diagnosis or treatment at present. Several agents have been implicated as potential causes. Non-infectious causes include mycotoxins (4), biogenic amines (5), and high-level dietary copper (6). The infectious agents that have been implicated include clostridium infection (7), chicken proventricular necrosis virus infection (8), and infectious bursal disease virus infection (9). The diagnosis of this disease relies solely on clinical manifestations and postmortem examination to observe the pathological changes in the glandular stomach and muscular stomach. Therefore, it is urgent to establish a replicable chicken model of glandular and muscular gastritis that is consistent with the clinical manifestations of this symptom. This model would greatly facilitate the prevention and treatment of this disease.

Acetic acid, polyethylene glycol, and ammonium chloride are common organic reagents that can cause damage to the gastrointestinal tract. Scientific investigations have substantiated that the ingestion of water containing polyethylene glycol and ammonium chloride can induce mucosal injury and ulceration in the glandular stomach and muscular stomach of chickens (10). Acetic acid has a corrosive effect that can directly damage the barrier function of the gastric and intestinal mucosa, leading to ulcer formation. It is commonly used in establishing animal models for ulcerative colitis and gastric ulcers (11, 12). Polyethylene glycol (PEG) is a polyether compound that cannot be digested or fermented. As a potential laxative, oral administration of PEG can increase the volume of fluid present in the gut (13, 14). Ammonium chloride is irritating and corrosive, causing damage to the gastrointestinal mucosa. Rhubarb, a traditional Chinese medicine categorized as bitter and cold, can cause damage to the gastric mucosal barrier function with long-term use. It is commonly used to establish animal models of chronic diarrhea and spleen deficiency syndrome (15). Previously, we conducted a comparative analysis on the efficacy of these reagents for modeling glandular and muscular gastritis in chickens, resulting in successful patent acquisition (16).

Currently, there is no published report on the establishment of a chicken model for this symptom. Therefore, we aim to establish an SPF chicken model of gastritis by combining various doses of polyethylene glycol, ammonium chloride, and rhubarb. Through screening for optimal modeling methods, our goal is to lay the foundation for further research into the mechanism of this syndrome and the development of improved treatment strategies for this type of syndrome.

## Materials and methods

### Establishment of the chicken model and grouping

Twenty-four 40-day SPF chickens were obtained from the animal center of Shandong Haotai Laboratory. The chickens underwent a 1-week acclimation period before the start of the experiment. The rhubarb used in this study was purchased from Jianlian Traditional Chinese Medicine Co., Ltd in Jinan, Shandong, China, and was authenticated by Prof. Zhongli Huang of the Shandong Academy of Agricultural Sciences. The rhubarb was soaked in distilled water at a ratio of 10 times its weight for 1 h, followed by decoction for 1.5 h. All filtrates were collected, and the residue underwent decoction two times. All filtrates were mixed and condensed to a concentration of 1.00 g/mL (crude drug). Solutions of polyethylene glycol, ammonium chloride, acetic acid, and rhubarb were prepared by adding them to water at appropriate concentrations prior to constructing the model. After a 7-day adaptation period, 24 chickens were randomly divided into four groups: six chickens in the M1 group were fed with polyethylene glycol (1.0%) and ammonium chloride (0.5%), six chickens in the M2 group were fed with acetic acid (2.0%) and rhubarb (750 mg/kg body weight), six chickens in the M3 group were fed with polyethylene glycol (1.0%) and rhubarb (750 mg/kg body weight), and six chickens served as a control group and were fed with sterilized water. The entire molding process lasted 14 days. At the end of the experiment, the chickens were intravenously injected with a sodium pentobarbital solution at a dosage of 50 mg/kg body weight and then euthanized by exsanguination. Each group was repeated three times. The animal experiment procedure was approved by the Ethics Committee of the Poultry Institute of Shandong Academy of Agricultural Sciences (SAAS; Approval number: JQS-2023-07).

### Measurement of clinical indicators

Clinical signs such as mental state, feces morphology, and color were recorded in detail during the experiment. The chickens were evaluated by semi-quantitative scoring based on their clinical symptoms (Table 1). The successful establishment of the glandular and muscular gastritis model was judged based on specific criteria. For instance, a sick chicken shows a poor mental state and has loose stools with a yellow hue mixed with undigested feed residues. The histological examination reveals hypertrophic glandular stomachs, thickened walls of the glandular stomach, edematous glandular gastric mucosa, and enlarged and prominent glandular gastric papillae. In severe cases, the glandular stomach may show blurred papillae or present ulcerative bleeding. The color of the muscular stomach stratum corneum is lightened to a pale yellow hue, with a rough and lackluster surface. Additionally, scattered ulcer foci of varying sizes and irregular shapes can be observed on its surface, with larger ulcerations appearing beneath the stratum corneum. No discernible pathological alterations are detected in other organs (16). The successful construction of chickens with glandular and muscular gastritis can be inferred if the aforementioned characteristics are observed in the model group. The high or low score reflects the severity of the disease. Body weight was measured at the start of modeling (day 1), midway through modeling (day 7), and at the conclusion of modeling (day 14) to calculate the effect of molding on weight gain rate. The body temperature of each group was measured at the start and end of modeling. At the end of the molding, the glandular and muscular stomachs and duodenum were removed for gross examination to assess bleeding and ulceration. Major organs, such as the spleen, bursa of Fabricius, and thymus, are weighed to calculate the relative organ weight.

**Table 1:** Semi-quantitative assessment of clinical manifestations of SPF chicken.

**Clinical manifestations**		**Score**
Mental state	Normal mental state	0
	Depressed spirits	1
	Emaciation, afraid of cold, and preferring warmth	3
	pale comb, yellow pigment loss of chicken feet (poor pigmentation of the shanks)	5
Morphology of stools	Formed manure	0
	Loose stools	1
	Loose stools with foam	3
	Loose stools with undigested feed	5
Glandular stomach injury	Intact gastric mucosa and clear gastric papilla	0
	Enlargement, gastric wall edema, and thickening, gastric papilla enlargement	3
	Severe enlargement, flushed gastric mucosa, flat gastric papilla	5
	Severe enlargement, blurred gastric papillae, slight ulcers, and bleeding	10
	Severe ulceration and bleeding in gastric mucosa	12
Muscular stomach injury	Complete stratum corneum, brown and yellow	0
	The color of the stratum corneum decreased, complete and smooth, and light yellow	3
	The color of the stratum corneum decreased, becoming uneven and thinner	5
	Ulcers of different sizes in the stratum corneum	10
	Severe ulceration and bleeding in stratum corneum	12

### Histopathological assays

Glandular and muscular stomachs, as well as duodenum, were collected immediately after chickens were euthanized. The organs were cut into small pieces with a scalpel and fixed in 4% paraformaldehyde for 2 days to denature and solidify the proteins in the tissue. The fixed tissue was trimmed, dehydrated, transparent, embedded, and sliced, and then the tissue sections were stained with hematoxylin and eosin (HE) using the standard protocols. Three random HE-stained sections were examined per specimen under a microscope (BX43F; Olympus, Tokyo, Japan).

### Serum cytokines detection

Blood samples were collected from the heart in vacuum tubes without anticoagulant for serum collection for subsequent experiments. The levels of IL-2, IL-4, and α-amylase in the serum of SPF chickens were detected using an ELISA kit (Jiyinmei Science and Technology Co Ltd. Wuhan, China) according to the manufacturer's instructions. The optical density (OD) at 450 nm was measured to obtain the sample values.

### 16S rRNA amplicon sequencing and sequence processing

After the experiment, cecal content samples were collected from chickens and immediately flash-frozen in liquid nitrogen before being stored at −80°C for subsequent bacterial DNA isolation. The total DNA of each sample was extracted using the CTAB method, as previously described (17). The quantity and quality of extracted DNA were analyzed using a NanoDrop Lite spectrophotometer (Thermo Scientific, USA). Then, using extracted DNA as a PCR template, the V3-V4 region of the 16S rRNA gene was amplified by PCR with universal primers forward 338 F and reverse 806 R. The reaction volume of PCR was 25 μL, containing 12.5 μL PCR mixed buffer, 1 μL of each primer (forward 338 F and reverse 806 R), 20 μg DNA, and dd H (2)O up to 25 μL. The PCR conditions were 98°C for 10 s, followed by 30 cycles of 98°C for 5 s, 50°C for 30 s, and 72°C for 30 s, with a final extension of 72°C for 10 min. The PCR products were purified by a Qiagen Gel Extraction Kit. The sequencing library was prepared using Illumina TruSeq^®^ DNA PCR-Free Sample Preparation Kit. Sequencing was performed on the Illumina NovaSeq platform for 2 × 250 bp paired-end sequencing.

The raw sequence data were processed using QIIME2 (18). Briefly, the primer sequencing in reads was removed by the Cutadapt pipeline. The amplicon sequence variant (ASV) and ASV table were generated by the DADA2 pipeline with default parameters (19). The taxonomic classification of each ASV was annotated by aligning the ASV's sequence to the SILVA database (release 132). The ASV classified into mitochondria and chloroplast were removed manually. The prediction of the metagenome function was performed using PICRUSt2 based on the ASV table (20).

### Statistical analysis

We conducted a rarefaction analysis of ASV numbers captured by increasing reads within each sample. The Shannon and Chao1 indexes were calculated using the QIIME diversity alpha function of QIIME2. The differences in the indexes between the two groups were analyzed using the non-parametric Wilcoxon rank sum test. Principal Coordinates Analysis (PCoA) and Non-metric Multidimensional Scaling (NMDS) analysis were performed using the Vegan package in R. For the ASV-to-AVS co-occurrence network, correlation coefficients were calculated using the SparCC algorithm (https://github.com/scwatts/fastspar). The statistical significance of correlations was calculated from 1,000 bootstrap iterations. The network was edited and visualized using Cytoscape v3.6.0 based on correlation coefficients. The property and module classification of the network were analyzed using the igraph package in R. The Mantel test was performed using the linkET package in R. The marker ASV or function of each group was detected using Linear discriminant analysis Effect Size (LefSe) analysis.

## Results

### Construction of gland and muscular gastritis model in SPF chicken

#### Changes in clinical symptoms in the SPF chickens

**Figure 1:**
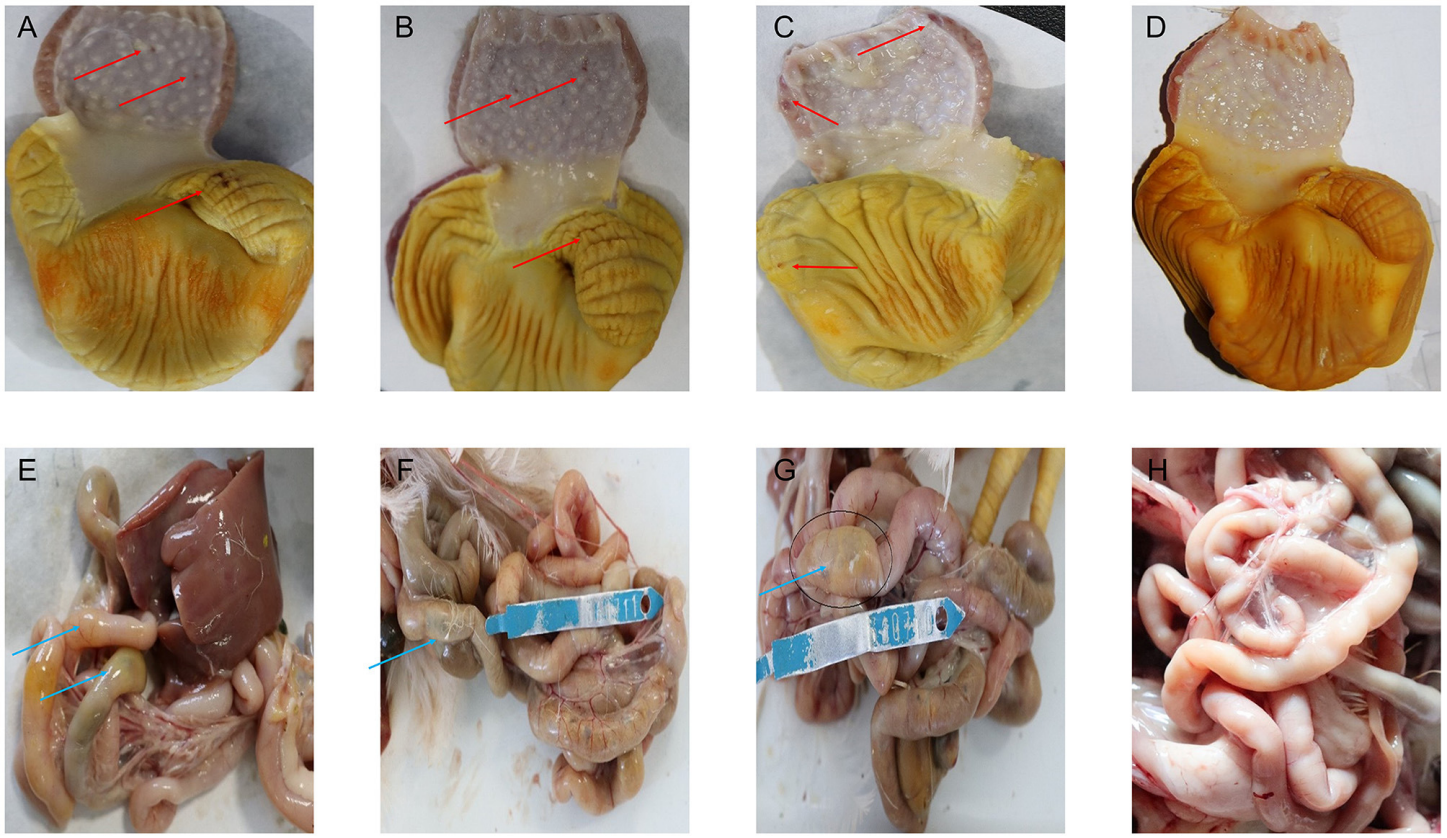
Pathological damage of the major organs. **(A–D)** Pathological results of glandular stomach and muscular stomach in chickens of each group. **(E–H)** Pathological results of duodenum in chickens of each group. The red arrow indicates the ulcers in the muscular stomach, and bleeds of gastric mucosa. The blue arrows indicate the intestinal distension.

**Figure 2:**
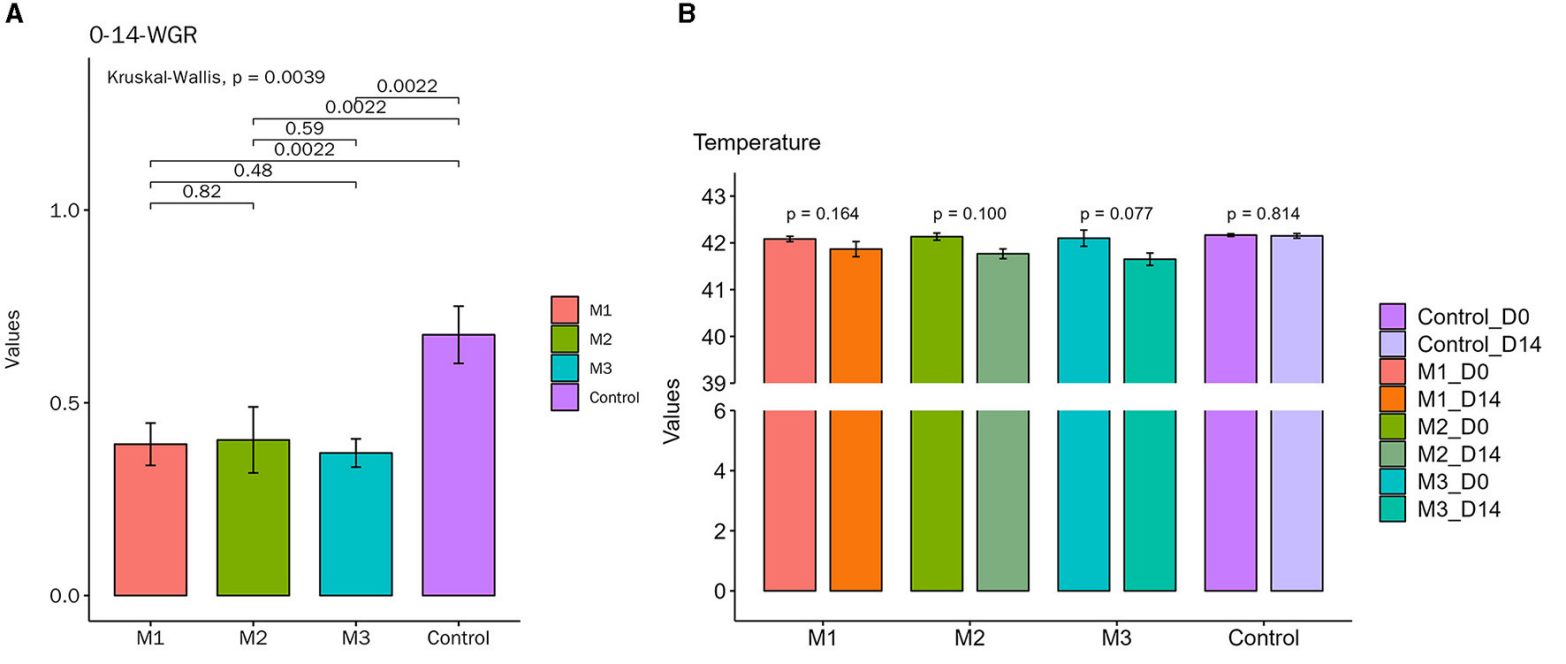
Body temperature and weight gain rate changes of chickens in all groups during the experiment. **(A)** Body temperature. The temperature remained within the normal range in the control group during the whole course of the experiment. The other groups showed a slight decrease in body temperature on day 14. **(B)** Weight gain rate. The weights increased gradually in each group during the whole course of the experiment. The weight gain rate of each experimental group was lower than that of the control group.

**Figure 3:**
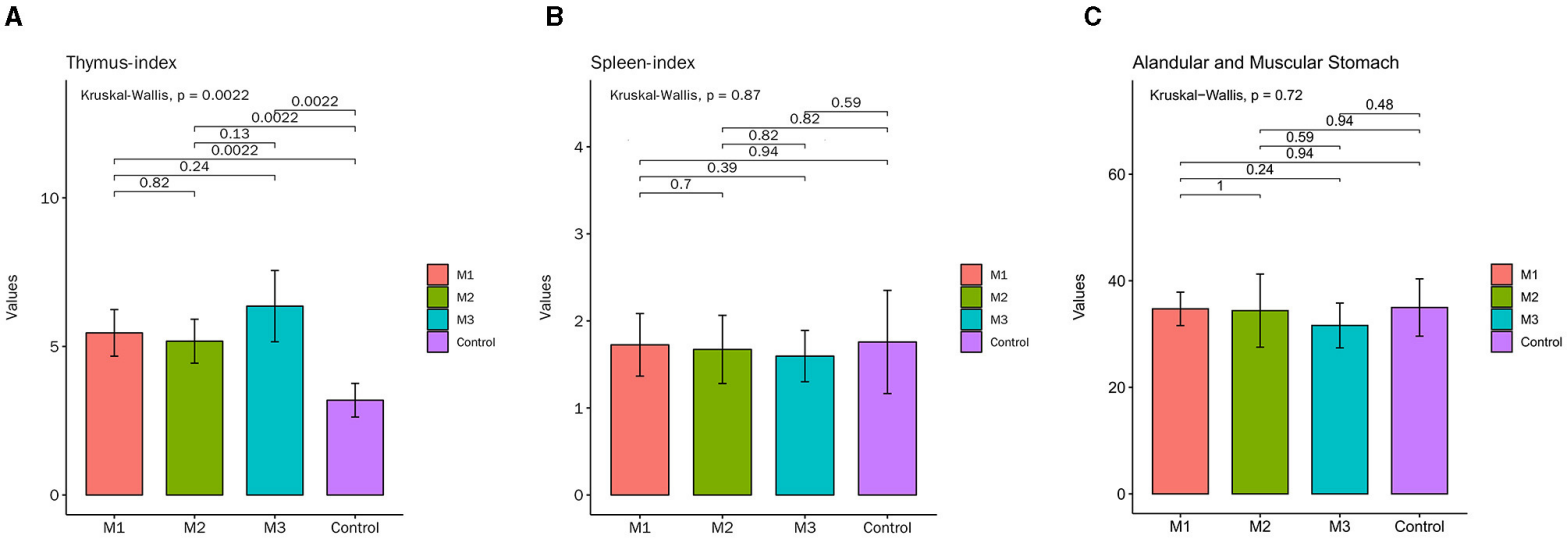
The major organ index of chickens in control and intervention groups. **(A)** Thymus-index. **(B)** Spleen-index. **(C)** Glandular and muscular stomach index.

On the 1st day of the experiment, all chickens were healthy, with a good mental state, clean and lustrous feathers, and normal body temperature. By day 14, the chickens in the control group remained healthy, while those in other experimental groups displayed a poor mental state and loose feces with undigested feed. Glandular and muscular stomach, as well as duodenal lesions, were found in each model group, characterized by an enlargement of the glandular stomach, ulcer in the muscular stomach, catarrhal inflammation, and bleeding of the gastric mucosa (Figure 1). The specific clinical symptoms of chickens in each group are provided in Table 2. According to this clinical symptom score, it was observed that the modeling success rate was 100% in the M2 and M3 groups, with more severe symptoms compared to the M1 group, which had an 86.33% modeling success rate and relatively mild symptoms overall. As shown in Figure 2, the body temperature of chickens in the control group remained within the normal range. In contrast, the body temperature of the chickens in the other groups was slightly lower on day 14. The weight gain of chickens in each group was gradual, but the weight gain rate of chickens in all model groups was significantly lower than that of the control group. The SPF chickens in the M3 group had the lowest weight gain rate (Figure 2A). Compared with the control group, the ratio of thymus and bursae of Fabricius weight to body weight in each model group was significantly increased, while the ratio of spleen weight to body weight decreased. The ratio of glandular and muscular stomach to body weight in the M2 and M3 groups was slightly lower than that in the control group, while it was slightly higher in the M1 group than that in the control group (Figure 3). The spleen index of the intervention group was lower than that of the control group, while the thymus index was higher than that of the control group (Figure 3). These results indicated that the three methods successfully established a chicken model with glandular and muscular gastritis, and the method of polyethylene glycol combined with rhubarb had the best effect.

**Table 2:** Clinical scoring statistics of SPF chickens.

**Score group**	**Mental state**	**Morphology of stools**	**Glandular stomach injury**	**Muscular stomach injury**	**Total score**
M1-1	1	0		10	11
M1-2	1	1	5	3	10
M1-3	3	0	0	10	13
M1-4	1	1	0	5	7
M1-5	1	1	0	0	2
M1-6	1	1	0	0	2
M2-1	3	5	5	10	26
M2-2	1	1	3	10	15
M2-3	1	0	3	5	9
M2-4	1	0	0	10	11
M2-5	3	1	5	10	19
M2-6	3	1	5	10	19
M3-1	5	5	12	5	27
M3-2	3	3	3	10	19
M3-3	5	5	3	12	25
M3-4	3	5	12	3	23
M3-5	5	3	5	10	23
M3-6	3	3	5	12	23
C-1	0	0	0	0	0
C-2	0	0	0	0	0
C-3	0	0	0	0	0
C-4	0	0	0	0	0
C-5	0	0	0	0	0
C-6	0	0	0	0	0

#### Changes of inflammatory cytokines in the serum of the SPF chickens

**Figure 4:**
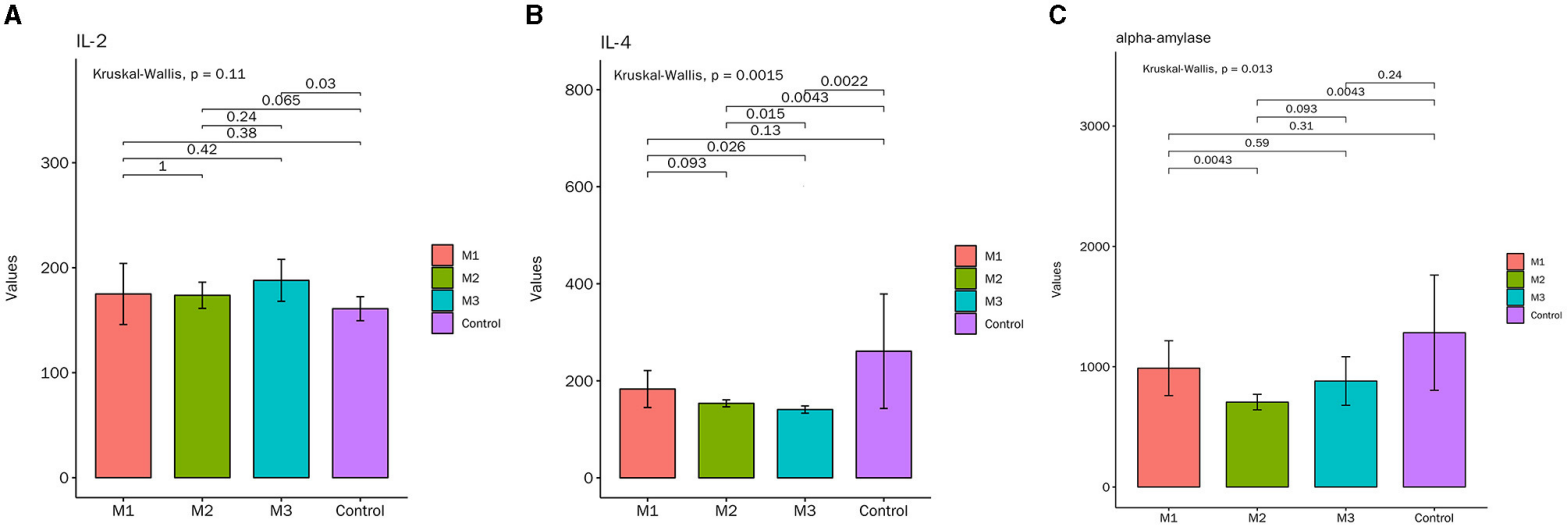
The contents of inflammatory cytokines in serum. **(A)** Interleukin (IL)-2, **(B)** IL-4, **(C)** α-amylase.

ELISA results indicated that the expression of IL-4 and α-amylase was decreased, while IL-2 was increased in the model groups (Figure 4). Furthermore, the intervention effect of the M2 and M3 groups was better than that of the M1 group. As shown in Figure 4B, the content of IL-4 in the M2 and M3 groups was significantly lower than in the control group, but there was no obvious difference between the two groups. The content of α-amylase in the M2 groups was significantly lower than that in the control group, while the change of α-amylase in the M1 group and M3 group was not significant (Figure 4C). These results suggested that the chicken model of glandular and muscular gastritis exhibits increased inflammation.

#### Histopathological changes of main organs in the SPF chickens

**Figure 5:**
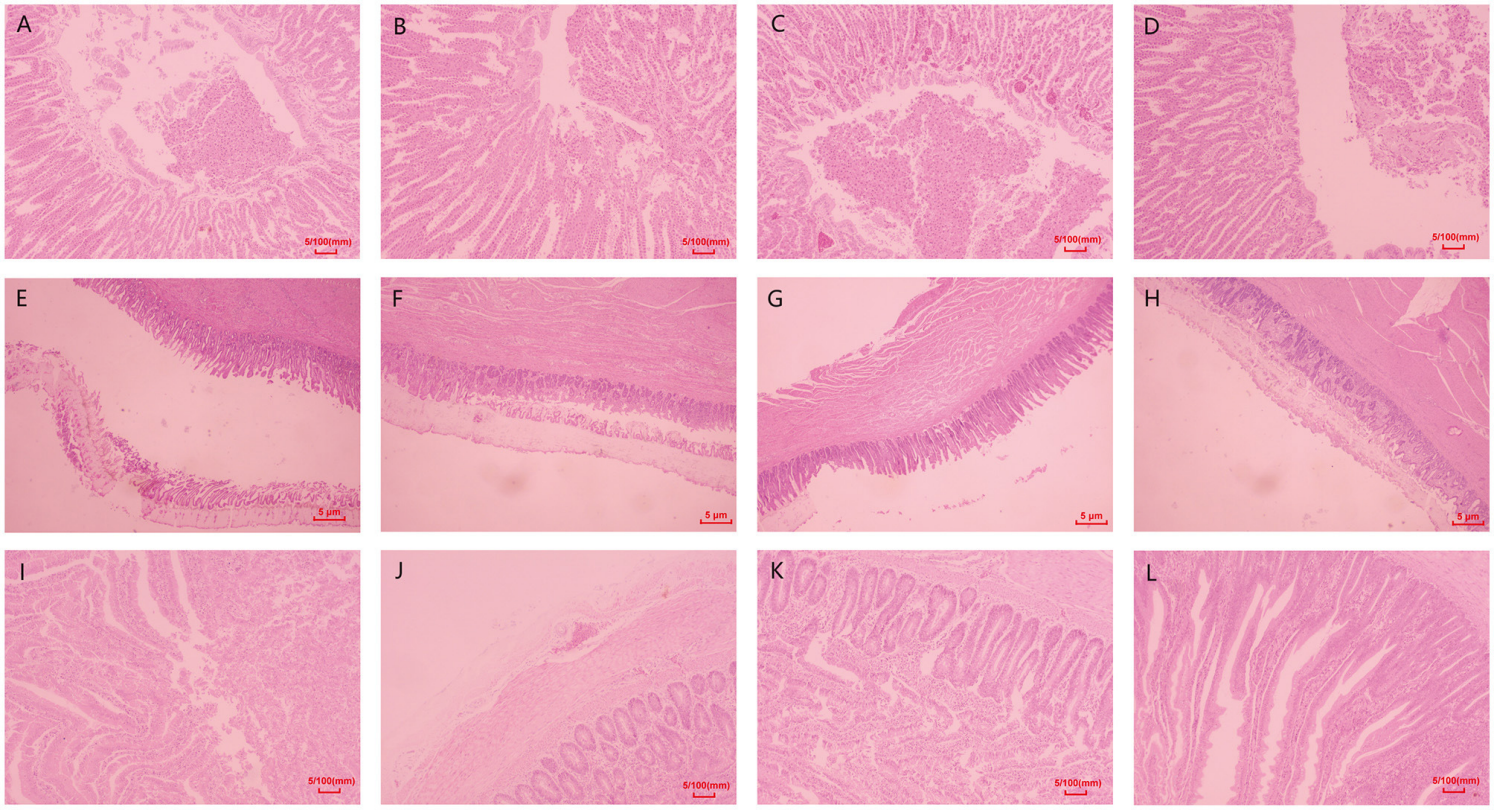
Histopathological changes of the major organs. **(A–D)** Histological results of the glandular stomach in chickens of each group. **(E–H)** Histological results of the muscular stomach in chickens of each group. **(I–L)** Histological results of duodenum in chickens of each group. The original magnification for A-H was 100×, and the original magnification for I-L was 40×.

Histopathological examination revealed that no obvious abnormalities were observed in the organs of chickens in the control group. Still, damages to the glandular and muscular stomach, as well as the duodenum, were observed in model groups (Figure 5).

Histopathological examination revealed no obvious abnormalities in the glandular and muscular stomach and duodenum of chickens in the control group. However, various degrees of lesions were observed in the organs of the model groups (Figure 5). Villus detachment was observed in the glandular stomach of all model groups, with specifically detected mucosal hemorrhage in the M3 group (Figures 5A–C). The M1 group exhibited edema and ulceration beneath the stratum corneum of the muscular stomach. Mild hemorrhage and inflammatory cell infiltration were observed beneath the stratum corneum of the muscular stomach in the M2 group. In contrast, extensive detachment of the stratum corneum was found in the muscular stomach of the M3 group (Figures 5E–G). Villus detachment was evident in both duodenum samples from the M1 and M3 groups, while serosal edema specifically occurred in duodenum samples from the M2 group (Figures 5I, G, K).

### The diversity of chicken gut microbiota in control and intervention groups

**Figure 6:**
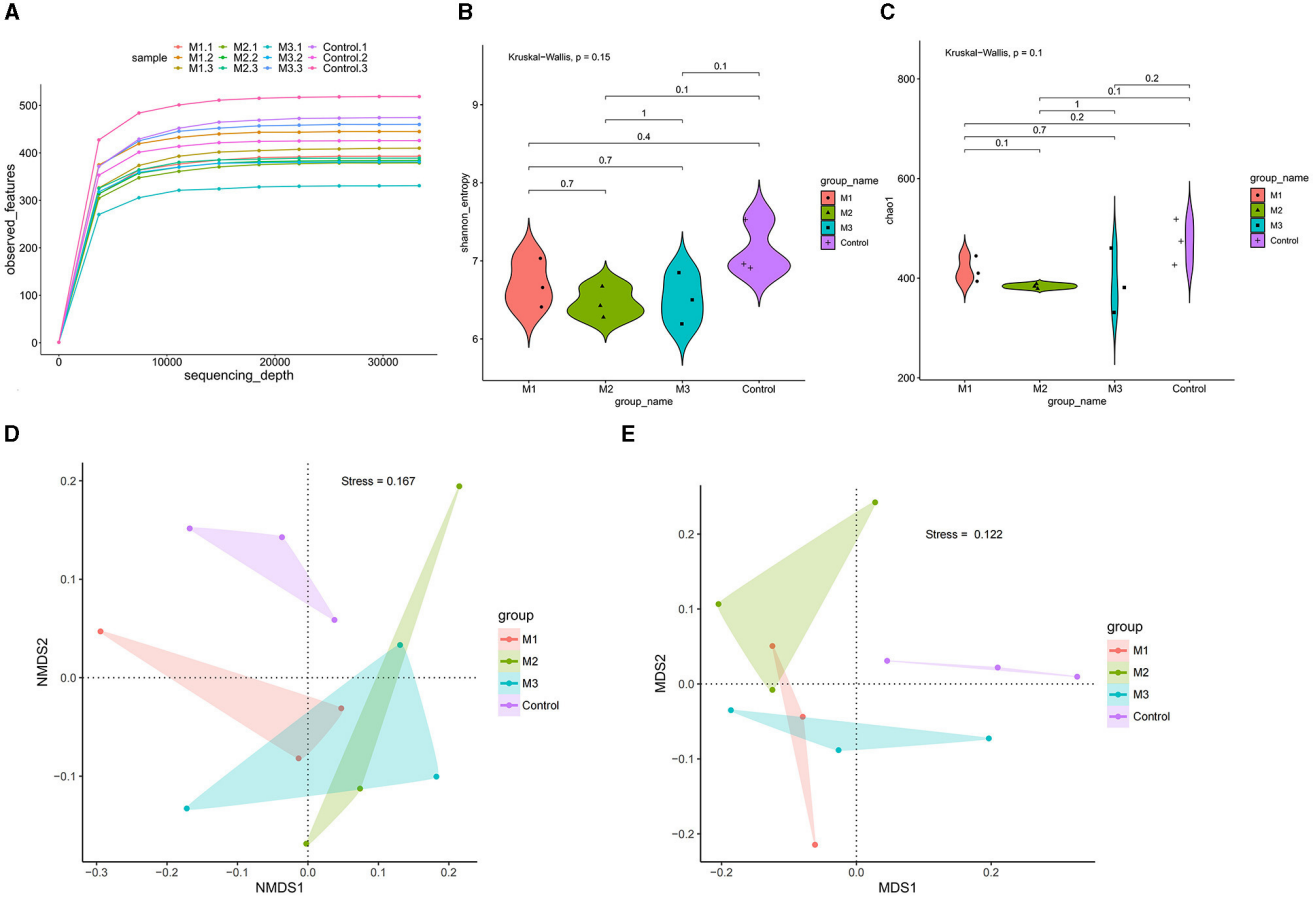
Gut microbial diversity in control and intervention groups. **(A)** Rarefaction curve analysis was used to show the sequencing quantity of each group. **(B)** Shannon-index by the non-parametric Wilcoxon rank sum test of alpha diversity. **(C)** Chao1-index by the non-parametric Wilcoxon rank sum test of alpha diversity. **(D)** PCoA of beta diversity of the phylum levels. **(E)** PCoA of beta diversity of the genus levels.

We conducted an amplicon sequencing analysis to assess the impact of various interventions on the gut microbiota of chickens. A total of 865,076 reads were obtained from all samples. After quality control, 725,226 clean reads were used to generate 1,655 ASVs. Rarefaction analysis revealed that all rarefaction curves reached a plateau (Figure 6A), indicating that the sequenced reads were sufficient to represent the number of ASVs in different samples.

Shannon (Figure 6B) and Chao1 (Figure 6C) indexes were used to characterize microbial diversity in different groups. The non-parametric Wilcoxon rank sum test showed a significant difference in diversity among the three model groups (*p* < 0.05). The Shannon and Chao1 indexes were highest in the M1 group, followed by the M3 and M2 groups. Compared with the control group, the Shannon and Chao1 indexes in all three intervention groups were significantly lower. These results indicated that the interventions reduced the diversity of chicken gut microbiota, and the effects of different interventions were divergent.

We performed dimension-reducing analysis at the phylum and genus levels to determine the variation in microbial community composition of chicken gut microbiota. As shown in Figures 6D, E, a group-based separation of microbial communities was observed between the control and intervention groups at both phylum and genus levels, suggesting different microbial community compositions between the control and intervention groups.

### The dominant microbial taxon in control and intervention groups

**Figure 7:**
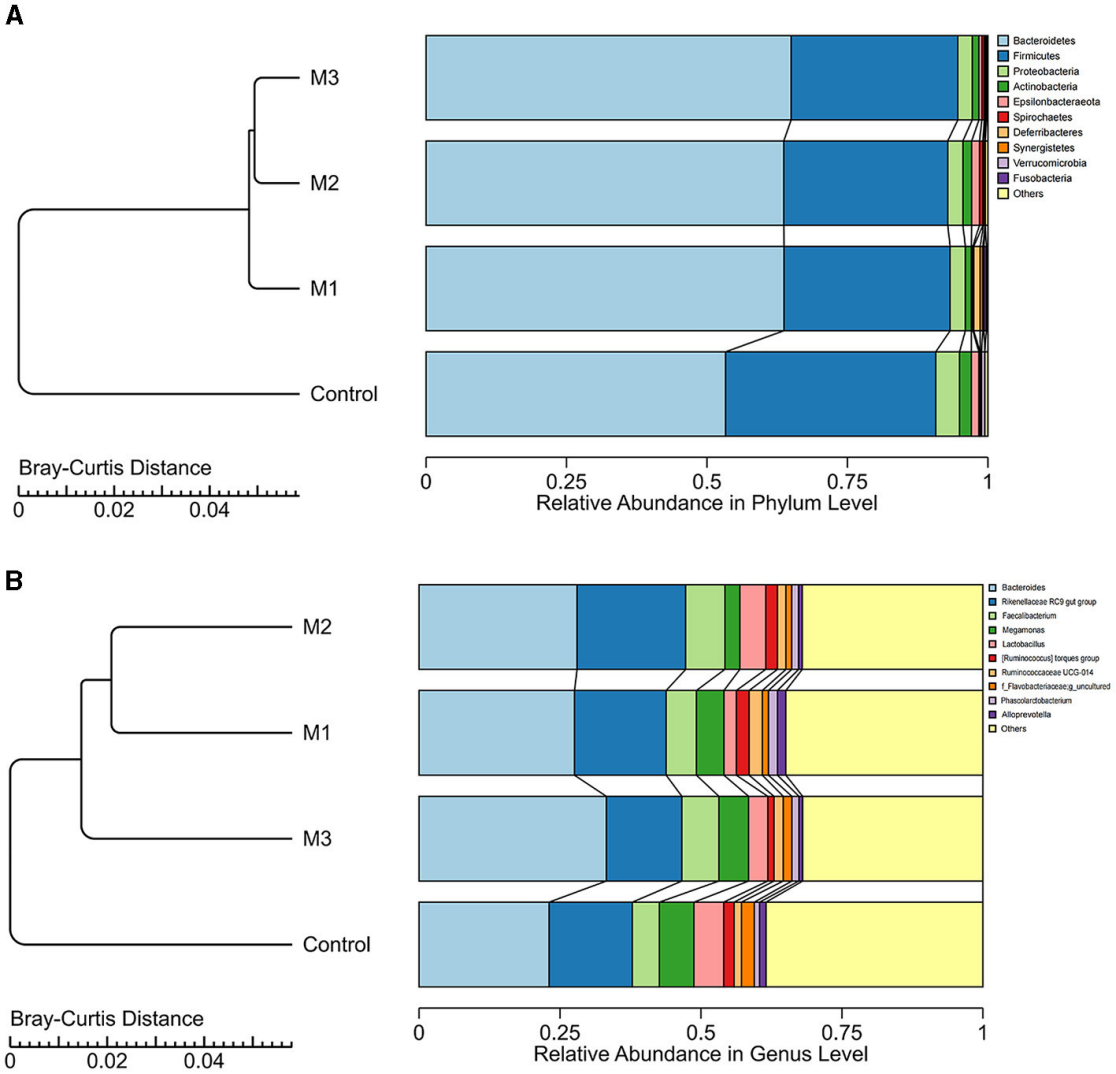
The dominant microbial taxa in control and intervention groups at phylum **(A)** and genus **(B)** levels.

A total of 16 phyla, 24 classes, 51 orders, 91 families, 188 genera, and 1,655 ASVs were identified in the control and intervention groups. We investigated the dominant microbial taxa at the phylum and genus levels. As shown in Figures 7A, B, the top 10 dominant phyla and genera in both control and intervention groups were consistent. The dominant phyla (average relative abundance > 1%) were Bacteroidetes, Firmicutes, Proteobacteria, and Actinobacteria. The dominant genera (average relative abundance > 1%) included *Bacteroides, Rikenellaceae RC9 gut group, Faecalibacterium, Megamonas, Lactobacillus, [Ruminococcus] torques group, Ruminococcaceae UCG-014*, and *Phascolarctobacterium*.

Based on the Bray-Curtis distance, three intervention groups and one control group were clustered into different clusters (Figure 7). The interventions led to an increase in the abundance of Bacteroidetes and a decrease in the abundance of Firmicutes, Proteobacteria, and Actinobacteria. At the genus level, four dominant genera showed increased abundance in all intervention groups, including *Bacteroides, Faecalibacterium, Ruminococcaceae UCG-014*, and *Phascolarctobacterium*. The abundance of *Megamonas* and *Lactobacillus* decreased after any interventions. We also observed the specific increased genera. For example, the abundance of *Rikenellaceae RC9 gut group* and *Ruminococcus torques group* increased in the M1 and M2 groups but decreased in the M3 group. These results indicate that the interventions altered the composition of dominated microbial taxa.

### Co-occurrence pattern in chicken gut microbiota

**Figure 8:**
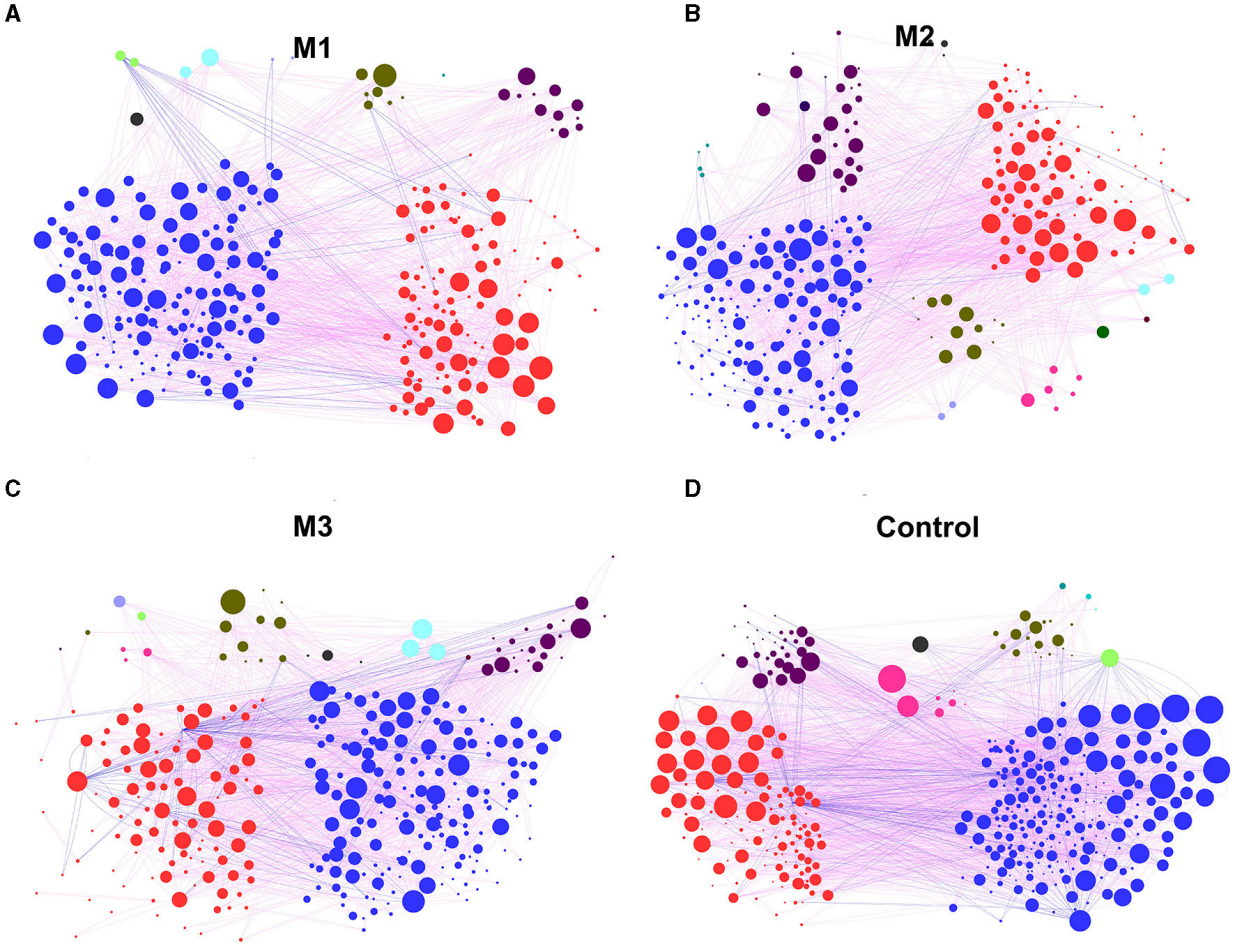
The co-occurrence pattern in chicken gut microbiota from one control group and three intervention groups. **(A)** M1 group. **(B)** M2 group. **(C)** M3 group. **(D)** Control group. The nodes represent the OTUs. The lines between nodes represent the Spearman correlation, and the color intensity represents the correlation coefficient (red, positive; blue, negative). The color of the nodes was based on different types of bacteria, and the size indicates the number of lines.

To determine the general effects of intervention on chicken gut microbiota associations, four networks were constructed for one control and three intervention groups, respectively (Figure 8). Based on the percentage of nodes in these networks, Bacteroidetes (37.09%, M1; 31.66%, M2; 32.22%, M3; and 28.17%, Control), Firmicutes (53.97%, M1; 49.22%, M2; 53.19%, M3; and 56.35%, Control) and Proteobacteria (3.97%, M1; 8.46%, M2; 6.99%, M3; and 7.87%, Control) were considered as core microbiota (Figure 8A). Compared to the control group, the clustering coefficient of the networks of the three intervention groups decreased by 3.5% (M1), 4.1% (M2), and 3.3% (M3), indicating that the interventions reduced the association of chicken gut microbiota (Figure 8B). For example, ASV_373 in the phylum Epsilonbacteraeota possessed the most connections in the control group network (30 edges) but was not linked to any taxon in the intervention group networks.

The average number of neighbors, clustering coefficient, network heterogeneity, and number of edges were used to reveal the differences between the control and intervention groups in the network. The results are shown in Supplementary Figure 1. All network indices in the intervention groups decreased compared to the control group. For example, the network heterogeneity in the intervention groups was 0.79 (M1), 0.85 (M2), and 0.86 (M3), which is lower than the 0.96 in the control group (Supplementary Figure 1C). The number of edges in the intervention groups was reduced to 871 (M1), 929 (M2), and 1,009 (M3; Supplementary Figure 1D).

### Specific taxon in chicken gut microbiota of control and intervention groups

**Figure 9:**
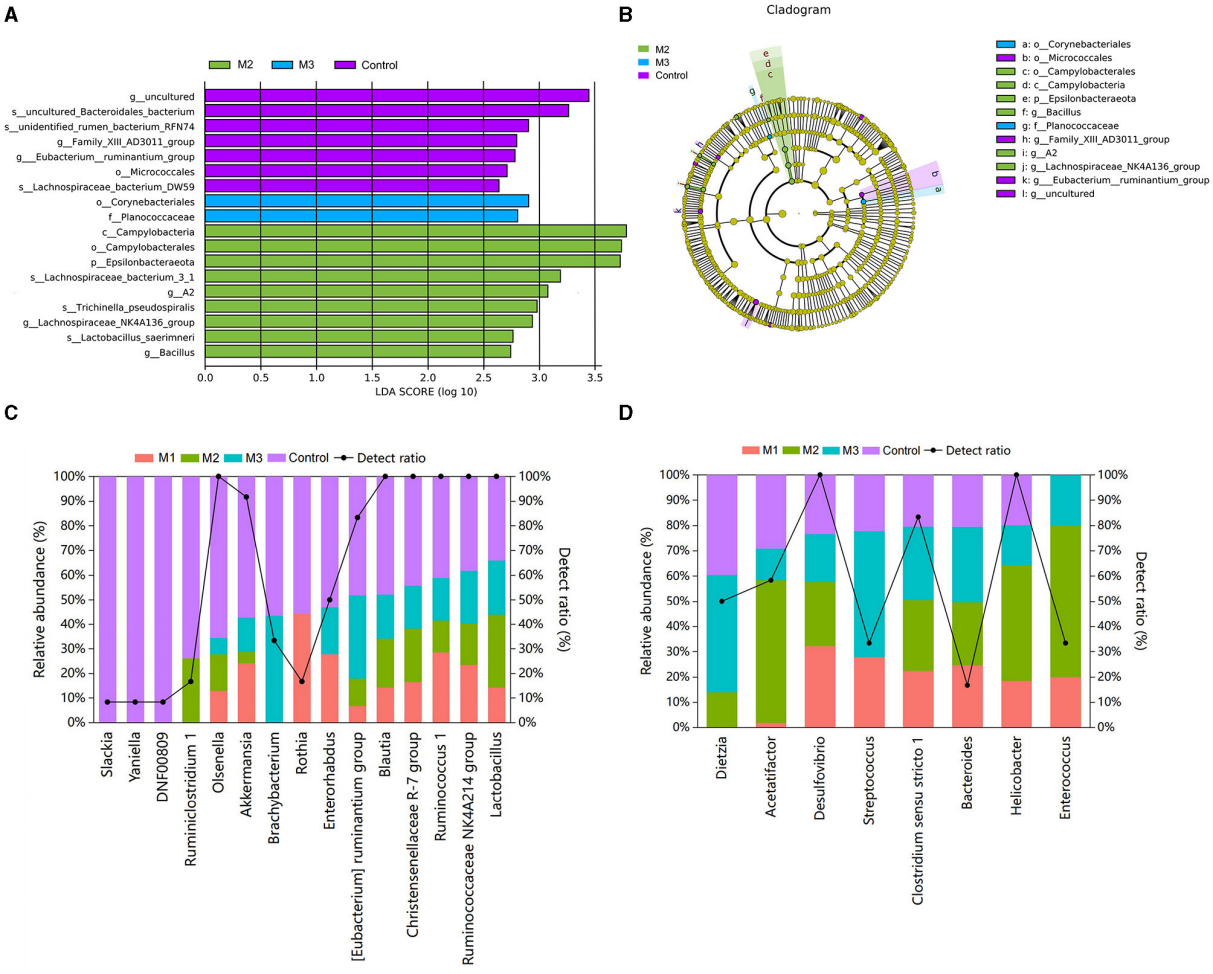
Specific taxon in chicken gut microbiota from control and intervention groups **(A)** and their taxonomic origin **(B)**. The relative abundances and detection ratios of probiotics **(C)** and pathogens **(D)**.

We performed a LEfSe analysis (Figure 9) to identify the specific taxon in the chicken gut microbiota of control and intervention groups. A total of 18 differential taxa were observed with LDA scores > 2 (Figure 9A). There were nine, two, and eight taxa enriched in M2, M3, and control groups, respectively. The taxonomic tree generated by LEfSe highlighted the taxonomic origin of taxa that were statistically differentiated among different groups (Figure 9B). We detected that the specific taxa enriched in the M2 group were mainly from Campylobacterales, Campylobacteraeota, and Epsilonbacteraeota. In the M3 group, one specific taxon was from Planococcaceae and Corynebacteriales. In the control group, the specific taxa were mainly from *Micrococcales*.

Furthermore, the probiotics and pathogens in the pooled data were identified based on previous studies (21–24). Fifteen probiotics were found in the chicken gut microbiota (Figure 9C). Among them, the *[Eubacterium] ruminantium group, Brachybacterium, Rothia*, and *Yaniella* were identified as specific taxa for the control group in the LEfSe analysis. The relative abundance of probiotics in the control group was higher than in the intervention groups (3.29%, M1; 5.46%, M2; 4.41%, M3; and 8.29%, Control). Notably, *Slackia, Yaniella*, and *DNF00809* probiotics were detected only in the control group.

Eight taxa were known to include potential poultry pathogens, and their relative abundances in the control group were lower than those in the intervention groups (29.15%, M1; 29.85%, M2; 34.30%, M3; and 24.37%, Control; Figure 9D). Among these potential pathogens, *Helicobacter* and *Dietzia* were specific taxa enriched in M2 and M3. The pathogen *Enterococcus* was only present in the intervention groups.

### The relationships between chicken gut microbiota and host indexes

**Figure 10:**
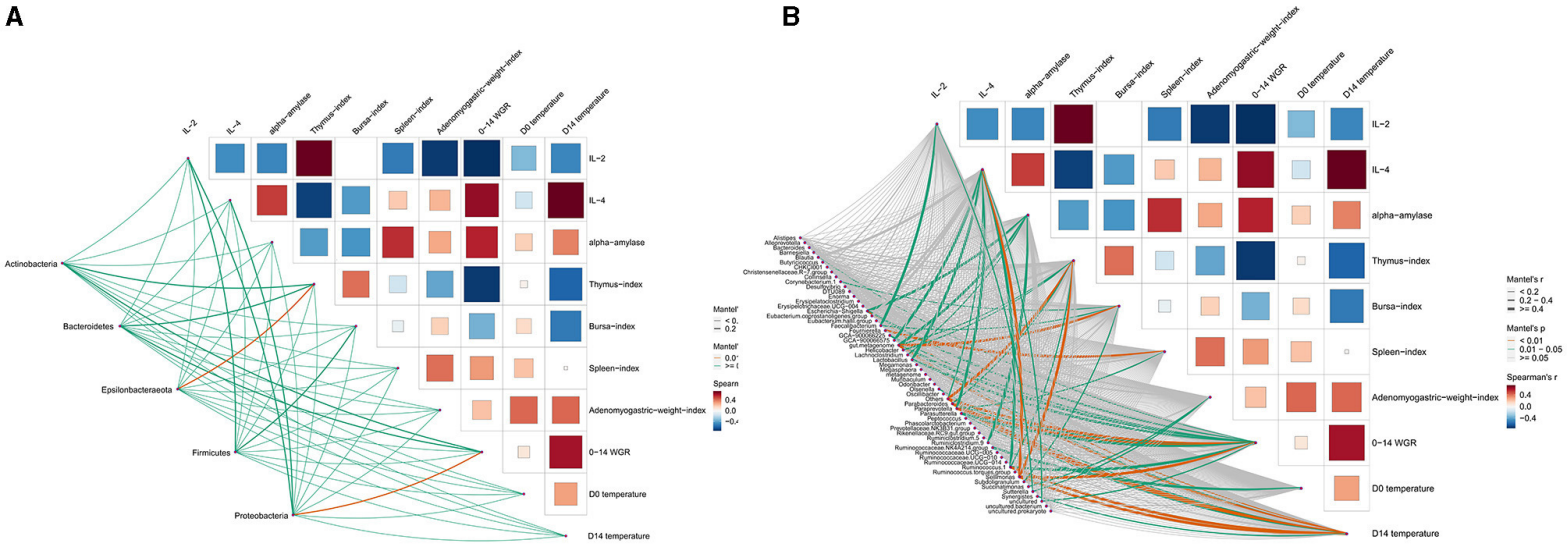
The Mantel test between chicken gut microbiota and host indexes. **(A)** Phylum level. **(B)** Genus level.

Spearman rank correlation was further employed to analyze the correlation between gut microbiota and host indexes (Figure 10). Among all phyla, two phyla showed a significant correlation (*p* < 0.05) with host indexes (Figure 10A). For example, Epsilonbacteraeota was related to the thymus index, and Proteobacteria was related to 0–14 WGR. At the genus level, 16 genera showed a significant correlation with host indexes, which suggests that these genera were associated with the host's health (Figure 10B). For example, the IL-4 of the control group was higher than that of the intervention groups, potentially resulting from the decreased abundance of *Blautia, Lactobacillus, Paraprevotella*, and *Parasutterella*. In addition, α-amylase was related to *Eubacterium.coprostanoligenes.group* and *GCA-900066575*. The Adenomyogastric weight index was related to *Bacteroides* and *Odoribacter*. The bursa index was related to *GCA-900066225*. The 7–14 WGR were related to *Faecalibacterium, Phascolarctobacterium*, and *Subdoligranulum*.

### The effect of intervention on chicken gut microbial function capacities

**Figure 11:**
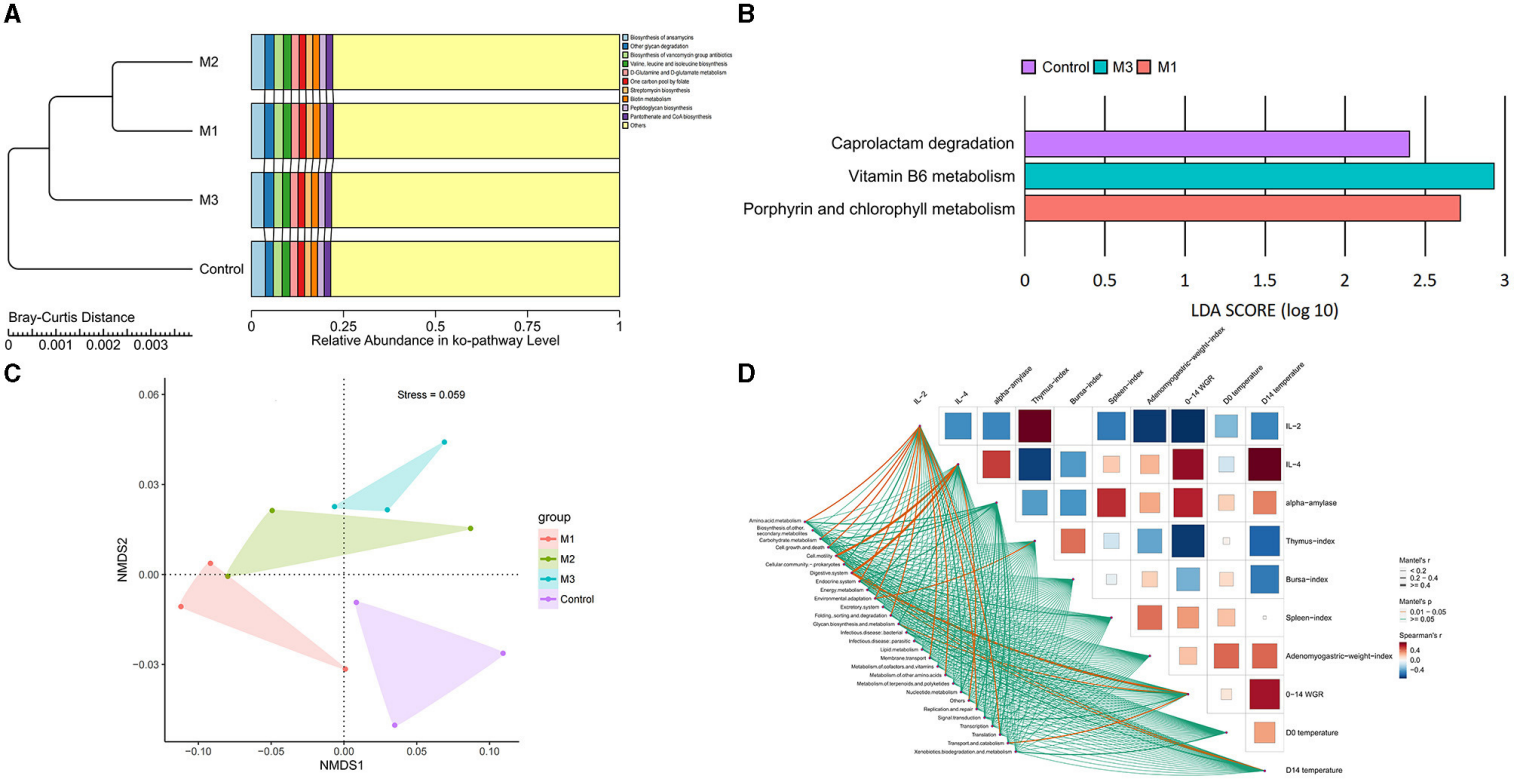
Characteristics of chicken gut microbial function in control and intervention groups. **(A)** The top 10 microbial function capacities based on their relative abundances. **(B)** The specific microbial functions in control and intervention groups. **(C)** NMDS analysis of microbial functions. **(D)** The correlation between the function capacities and host indexes.

The most active pathways in all groups were annotated to carbohydrate metabolism, membrane transport, replication and repair, translation, and amino acid metabolism, accounting for 48.76–48.96% (Figure 11A). To illustrate the functional alterations of chicken gut microbiota after interventions, PCoA analysis was performed. The results are shown in Figure 11C. We observed the distinct microbial function capacities associated with the control and intervention groups. Based on LEfSe analysis, three differential active function capacities were observed with LDA scores > 2 (Figure 11B). Specifically, the control group showed more active degradation of caprolactam, a more active metabolism of vitamin B6 was seen in M3, and a more active metabolism of porphyrin and chlorophyll was observed in M1.

Further, the correlation between the function capacities and host indexes was analyzed (Figure 11D). Thirteen items were found to be related to host indexes (*p* < 0.05), among which three pathways showed a strong correction (*R* >0.4). Cell motility and the digestive system were associated with IL-4, while the digestive system was linked to 0–14 WGR.

## Discussion

Glandular and muscular gastritis is a transmissible disease that affects commercial chickens. It is characterized by the enlargement and ulceration of the glandular and muscular stomachs, poor feed conversion, poor uniformity, decreased body weight, and loose stools with undigested feed (5). The precise etiology of glandular and muscular gastritis remains unknown. It is urgent to establish a replicable chicken model of glandular and muscular gastritis consistent with the clinical manifestations of this symptom to help prevent and treat this disease.

In this study, we explored various methods for establishing SPF chicken models with glandular and muscular gastritis. Our findings reveal that the levels of IL-4 and α-amylase in the successfully constructed model chickens are reduced, while the contents of serum pro-inflammatory cytokines IL-2 are increased, indicating that this disease may enhance the inflammatory response and reduce feed conversion in diseased chickens. Gastritis, a common gastropathy often accompanied by inflammation, is evaluated for severity through the expression of inflammatory markers. IL-4 has been observed to play an inhibitory role in the expression and release of inflammatory cytokines. These inflammatory responses could also promote the infiltration of inflammatory cells and cause injury to the glandular and muscular stomach. Additionally, the establishment of the model significantly reduced the growth performance of chickens, which may be related to a reduction in feed conversion rate caused by injuries to the glandular and muscular stomachs in sick chickens. Amylase is an exocrine enzyme secreted by the pancreas and salivary glands, catalyzing the conversion of starch and glucose into smaller components (25). α-amylase is an important digestive enzyme component in the body, playing a vital role in intracellular energy transfer, muscle contraction, and ATP regeneration. Its activity also has an important influence on feed digestion, absorption, and utilization (26). The decrease of α-amylase may correspond to the slow growth in sick chickens. In clinical settings, serum amylase has been used as a biochemical marker for both acute and chronic pancreatitis to provide diagnostic support (27), but its relationship with gastritis has hardly been reported. Given the multifaceted nature of α-amylase, we need to delve deeper into its potential relationship with chicken gastritis.

In this study, the microbial communities of the model groups exhibited distinct differences compared to those of the control group. Different treatments have different effects on the microbial diversity of chicken intestines, but one commonality is that the microbial diversity of chicken intestines is reduced after establishing the model. Through the *t*-test, we identified significant differences in flora between the model groups and the control. Notably, most of the genera with significant differences were found to be significantly reduced in the model group, including *Acetatifactor, Tuzzerella, Prevotellaceae*, and *[Eubacterium]_brachy_group*. *Acetatifactor* is a strictly anaerobic Gram-positive bacterial genus from the Lachnospiraceae family, which is closely related to bile acid metabolic (28) and short-chain fatty acid metabolism (29). It has also been reported that *Acetatifactor* undergoes significant changes in colitis, and the regulation of acetylation factors plays a crucial role in relieving colitis (30). *Prevotellaceae* is a probiotic in the intestine that helps to break down proteins and carbohydrates. It has been reported that certain intestinal microflora and their metabolites, including *Prevotellaceae*, play important roles in maintaining intestinal barrier integrity and intestinal homeostasis (31). *Eubacterium* was first proposed by Prévot in 1938 to describe a group of beneficial bacteria isolated from human feces, which have the functions of antagonizing nutritional organisms and maintaining the ecological balance of intestinal microorganisms. Eubacterium species are important for gut health and have also shown negative correlations with inflammatory markers such as IL-2 and C-reactive protein (32). In this study, the changes in intestinal flora in model chickens also correspond to an increase in inflammation caused by glandular and muscular gastritis. Furthermore, the relative abundance of known species other than the top 10 in the mean abundance ranking was relatively high in the control group (more than 30% at the genus level), suggesting that there may be more unknown species to be discovered.

Overall, this research strictly adhered to experimental standards throughout the entire procedure. However, there is still room for improvement in some aspects. Firstly, the number of SPF chickens used in the experiment was not large, so we plan to use a larger number of experimental animals to carry out experiments in order to further determine the repeatability and stability of model construction and the possibility of measured indicators as biomarkers for glandular and muscular gastritis. Additionally, we will comprehensively apply proteomics, metabonomics, and other techniques in the next experiment to explore the pathogenesis of this disease more clearly.

## Conclusions

All model groups of chickens exhibited typical symptoms of glandular and muscular gastritis through the three modeling methods described in the text, indicating successful reproduction of the chicken model. After establishing the SPF chicken model of glandular and muscular gastritis, it mainly caused slow weight gain, decreased serum IL-4 and α-amylase levels, ulceration and bleeding in the glandular and muscular stomach, as well as disturbances in gut microbiota. Through comprehensive comparisons of model construction using various methods, it was determined that the SPF chicken model constructed using polyglycol combined with rhubarb was more consistent with glandular and muscular gastritis syndrome. The successful establishment of this model can help improve studies on the prevention and treatment of glandular and muscular gastritis.

## Data availability statement

The datasets presented in this study can be found in online repositories. The names of the repository/repositories and accession number(s) can be found at: https://www.ncbi.nlm.nih.gov/, PRJNA1017104.

## Ethics statement

The animal study was approved by Laboratory Animal Ethics Commission of the Poultry Institute of Shandong Academy of Agricultural Science. The study was conducted in accordance with the local legislation and institutional requirements.

## Author contributions

ZY: Writing – original draft. SY: Conceptualization, Methodology, Writing – review & editing. SL: Project administration, Writing – review & editing. ZZ: Supervision, Writing – review & editing. YL: Formal analysis, Writing – review & editing. BY: Methodology, Writing – review & editing. YY: Conceptualization, Writing – review & editing. SS: Investigation, Writing – review & editing. RZ: Writing – review & editing. ZH: Conceptualization, Writing – original draft.

## Supplementary material

Supplementary Figure 1 Comparative analysis of network properties among groups.

## References

[1] Marusak RA West MA Davis JF Fletcher OJ Guy JS. Transmissible viral proventriculitis identified in broiler breeder and layer hens. Avian Dis. (2012) 56:757–9. 10.1637/10216-042412-Case.1 23397852

[2] Guy JS West MA Fuller FJ Marusak RA Shivaprasad HL Davis JL. Detection of chicken proventricular necrosis virus (R11/3 virus) in experimental and naturally occurring cases of transmissible viral proventriculitis with the use of a reverse transcriptase-PCR procedure. Avian Dis. (2011) 55:70–5. 10.1637/9586-102110-Reg.1 21500639

[3] Ohh MH Kim S Pak SC Chee KM. Effects of dietary supplementation with astaxanthin on histamine induced lesions in the gizzard and proventriculus of broiler chicks. Asian-Australas J Anim Sci. (2016) 29:672–8. 10.5713/ajas.15.1020 26954210 PMC4852255

[4] Dinev I. Enzootic outbreak of necrotic gastritis associated with *Clostridium perfringens* in broiler chickens. Avian Pathol. (2010) 39:7–10. 10.1080/03079450903431382 20390530

[5] Pantin-Jackwood MJ Brown TP Huff GR. Proventriculitis in broiler chickens: immunohistochemical characterization of the lymphocytes infiltrating the proventricular glands. Vet Pathol. (2004) 41:641–8. 10.1354/vp.41-6-641 15561672

[6] Smiałek M Gesek M Dziewulska D Koncicki A. Relationship between chicken proventricular necrosis virus prevalence and transmissible viral proventriculitis in broiler chickens in Poland. Pol J Vet Sci. (2021) 24:385–91. 10.24425/pjvs.2021.138729 34730315

[7] Smiałek M Gesek M Dziewulska D Niczyporuk JS Koncicki A. Transmissible viral proventriculitis caused by chicken ProVentricular necrosis virus displaying serological cross-reactivity with IBDV. Animals. (2020) 11:10008. 10.3390/ani11010008 33374720 PMC7822447

[8] Dorner JW Cole RJ Lomax LG Gosser HS Diener UL. Cyclopiazonic acid production by *Aspergillus flavus* and its effects on broiler chickens. Appl Environ Microbiol. (1983) 46:698–703. 10.1128/aem.46.3.698-703.1983 6416167 PMC239337

[9] Barnes DM Kirby YK Oliver KG. Effects of biogenic amines on growth and the incidence of proventricular lesions in broiler chickens. Poult Sci. (2001) 80:906–11. 10.1093/ps/80.7.906 11469653

[10] Zhongli H Shuqian L Minxun S Feng M Fu J Zhao Z. A novel approach for the rapid construction of a chicken spotted kidney model (Chinese). Shandong Province: CN105582553B (2019).

[11] Shao S Wang D Zheng W Li X Zhang H Zhao D. A unique polysaccharide from *Hericium erinaceus* mycelium ameliorates acetic acid-induced ulcerative colitis rats by modulating the composition of the gut microbiota, short chain fatty acids levels and GPR41/43 respectors. Int Immunopharmacol. (2019) 71:411–22. 10.1016/j.intimp.2019.02.038 31059977

[12] Kim YS Nam Y Song J Kim H. Gastroprotective and healing effects of *Polygonum cuspidatum* root on experimentally induced gastric ulcers in rats. Nutrients. (2020) 12:82241. 10.3390/nu12082241 32727104 PMC7468921

[13] de Maistre S Vallée N Gaillard S Duchamp C Blatteau JE. Stimulating fermentation by the prolonged acceleration of gut transit protects against decompression sickness. Sci Rep. (2018) 8:10128. 10.1038/s41598-018-28510-x 29973647 PMC6031626

[14] Shabani M Kefayati M Hassanian-Moghaddam H Zamani N McDonald R. Complications and hospital stay after endoscopic retrieval of drug baggies in body stuffers: an observational prospective study. Sci Rep. (2021) 11:5359. 10.1038/s41598-021-84898-z 33686170 PMC7940431

[15] Peng Y Wu C Yang J Li X. Gut microbial diversity in rat model induced by rhubarb. Exp Anim. (2014) 63:415–22. 10.1538/expanim.13-0104 25048267 PMC4244290

[16] Zhongli H Shuqian L Zengcheng Z Liu Y Yin B Yang S. A rapid method to construct a chicken model of gland and muscular gastritis (Chinese). Shandong Province: CN112616777B (2022).

[17] Honoré-Bouakline S Vincensini JP Giacuzzo V Lagrange PH Herrmann JL. Rapid diagnosis of extrapulmonary tuberculosis by PCR: impact of sample preparation and DNA extraction. J Clin Microbiol. (2003) 41:2323–9. 10.1128/JCM.41.6.2323-2329.2003 12791844 PMC156509

[18] Bolyen E Rideout JR Dillon MR Bokulich NA Abnet CC Al-Ghalith GA. Reproducible, interactive, scalable and extensible microbiome data science using QIIME 2. Nat Biotechnol. (2019) 37:852–7. 10.1038/s41587-019-0209-9 31341288 PMC7015180

[19] Callahan BJ McMurdie PJ Rosen MJ Han AW Johnson AJ Holmes SP. DADA2: high-resolution sample inference from Illumina amplicon data. Nat Methods. (2016) 13:581–3. 10.1038/nmeth.3869 27214047 PMC4927377

[20] Douglas GM Maffei VJ Zaneveld JR Yurgel SN Brown JR Taylor CM. PICRUSt2 for prediction of metagenome functions. Nat Biotechnol. (2020) 38:685–8. 10.1038/s41587-020-0548-6 32483366 PMC7365738

[21] Rychlik I. Composition and function of chicken gut microbiota. Animals. (2020) 10:10103. 10.3390/ani10010103 31936291 PMC7022619

[22] Yang Q Liu J Wang X Robinson K Whitmore MA Stewart SN. Identification of an intestinal microbiota signature associated with the severity of necrotic enteritis. Front Microbiol. (2021) 12:703693. 10.3389/fmicb.2021.703693 34489892 PMC8418326

[23] He Q Zhang Y Ma D Zhang W Zhang H. *Lactobacillus casei* Zhang exerts anti-obesity effect to obese glut1 and gut-specific-glut1 knockout mice via gut microbiota modulation mediated different metagenomic pathways. Eur J Nutr. (2022) 61:2003–14. 10.1007/s00394-021-02764-0 34984487

[24] Rom O Liu Y Liu Z Zhao Y Wu J Ghrayeb A. Glycine-based treatment ameliorates NAFLD by modulating fatty acid oxidation, glutathione synthesis, and the gut microbiome. Sci Transl Med. (2020) 12:aaz2841. 10.1126/scitranslmed.aaz2841 33268508 PMC7982985

[25] Peng YF Goyal H Lin H Liu DC Li L. Serum amylase activity altered by the ABO blood group system in Chinese subjects. J Clin Lab Anal. (2019) 33:e22883. 10.1002/jcla.22883 30938472 PMC6595472

[26] Furné M García-Gallego M Hidalgo MC Morales AE Domezain A Domezain J. Effect of starvation and refeeding on digestive enzyme activities in sturgeon (*Acipenser naccarii*) and trout (*Oncorhynchus mykiss*). Comparat Biochem Physiol A Mol Integr Physiol. (2008) 149:420–5. 10.1016/j.cbpa.2008.02.002 18328757

[27] Madole MB Iyer CM Madivalar MT Wadde SK Howale DS. Evaluation of biochemical markers serum amylase and serum lipase for the assessment of pancreatic exocrine function in diabetes mellitus. J Clin Diagn Res. (2016) 10:bc01–bc4. 10.7860/JCDR/2016/23787.8900 28050357 PMC5198310

[28] Wang K Xu X Shan Q Ding R Lyu Q Huang L. Integrated gut microbiota and serum metabolomics reveal the protective effect of oleanolic acid on liver and kidney-injured rats induced by *Euphorbia pekinensis*. Phytother Res. (2022) 2022:ptr.7673. 10.1002/ptr.7673 36426741

[29] Pfeiffer N Desmarchelier C Blaut M Daniel H Haller D Clavel T. *Acetatifactor muris* gen. nov, sp nov, a novel bacterium isolated from the intestine of an obese mouse. Archiv Microbiol. (2012) 194:901–7. 10.1007/s00203-012-0822-1 22659832

[30] Li T Gao X Yan Z Wai TS Yang W Chen J. Understanding the tonifying and the detoxifying properties of Chinese medicines from their impacts on gut microbiota and host metabolism: a case study with four medicinal herbs in experimental colitis rat model. Chin Med. (2022) 17:118. 10.1186/s13020-022-00673-w 36195889 PMC9533630

[31] Wang G Sun S Wu X Yang S Wu Y Zhao J. Intestinal environmental disorders associate with the tissue damages induced by perfluorooctane sulfonate exposure. Ecotoxicol Environ Saf. (2020) 197:110590. 10.1016/j.ecoenv.2020.110590 32283409

[32] Mukherjee A Lordan C Ross RP Cotter PD. Gut microbes from the phylogenetically diverse genus Eubacterium and their various contributions to gut health. Gut Microbes. (2020) 12:1802866. 10.1080/19490976.2020.1802866 32835590 PMC7524325

