# Supplementary figures and images for: Comparison of gut microbiota immunity and pathology in specific-pathogen-free chickens with glandular and muscular gastritis using different methods
Source: Front Vet Sci. 2024 Jun 3;11:1343768. doi: 10.3389/fvets.2024.1343768 (PMC11180906; doi:10.3389/fvets.2024.1343768)

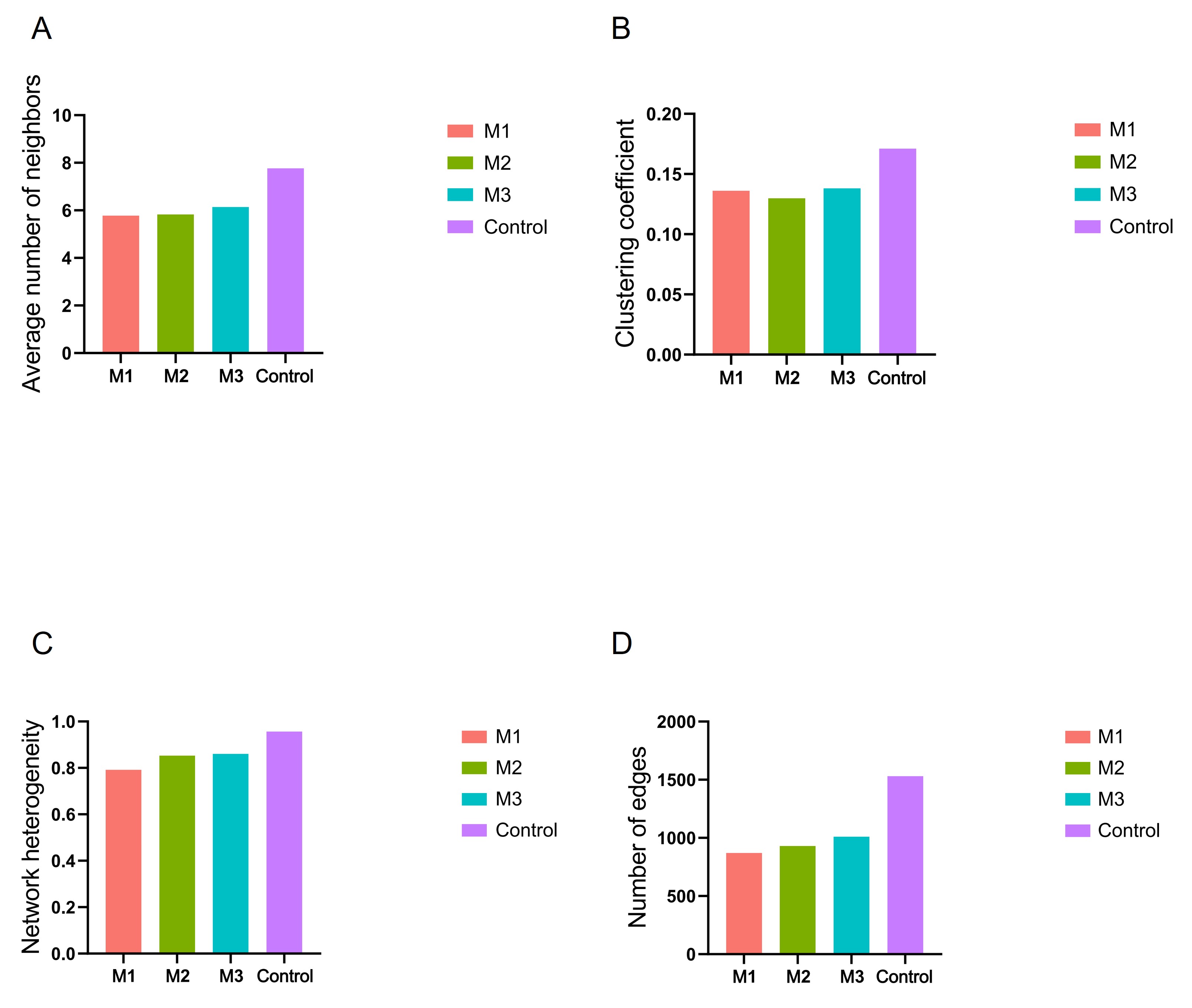

Supplement: Supplementary Figure 1 — Comparative analysis of network properties among groups. [file Image_1.jpg]
